
JOURNAL INFORMATION
==============================
NLM Title Abbreviation: Buffalo Med J Mon Rev Med Surg Sci
NLM Journal ID: 0004276

The Buffalo Medical Journal and Monthly Review of Medical and Surgical Science

Publisher: Biomedical Journal Digitization Project

ARTICLE INFORMATION
==============================
PMCID: PMC8932448
PMID: 35376945
Article version: 1
Subjects: Editorial Department

New York State Inebriate Asylum

Print publication date: 1858 Dec
Volume: 14
Issue: 7
First page: 440
Last page: 441
License: This work is free of known copyright restrictions. For more information, please see PMC Back Issue Digitization.
License URL: https://creativecommons.org/publicdomain/mark/1.0/

==============================

attempt at a "*Relief Karte*" of the more salient features of a profession in the honest exercise of which I hope soon to find every member of your class.

I have the honor to be,
Gentlemen,
Your obedient servant,
THEOPH. MACK.

Messrs. GEO. A. MCDONELL,
LUCIEN A. DAMAINVILLE,
JOSEPH W. ROBINSON,
NEWTON L. BATES, and
FRANK HODGE,
Members of Committee of Class, Med. Dep't University of Buffalo.

---

*New York State Inebriate Asylum.*—The corner stone of this institution was laid on the 24th of September last, with the impressive Masonic ceremonies. The Grand Master of the State of New York, and many other high dignitaries of the order, officiated in the exercises. Speeches were made by the Hon. Edward Everett, Hon. B. F. Butler, Hon. D. S. Dickinson, Dr. John W. Francis, and the Rev. Dr. Bellows. A poem was also read by Alfred B. Street, Esq. These, with the Masonic ceremonies and speeches from some of the order, made the exercises extremely interesting and impressive. We learned from one who had the good fortune to be present, that the speech by Everett was one of his happiest efforts. A speech from this great man alone, would give interest to such exercises, and the happy efforts by the remaining orators, combined to make the affair most satisfactory and successful. The distress, both mental and physical, to which the vice of intemperance will inevitably lead, is brought more forcibly to the mind of no class of men, than to the physician. We know the terrible diseases to which it gives rise; we see the misery and desolation which is brought into a virtuous home by the demon of drink, where it is concealed from all the world beside. The sacred ties of the family physician lead the unhappy to unburden their heart, to lay bare all their woe, and to ask him, almost as a superior being, if there be no remedy. A man, and a man of intellect and education, can see himself the cause of incalculable unhappiness, can wish and pray to have strength to resist, yet the hold which a habit of stimulating obtains upon the organism is of such a character, that it requires the strongest will to give up a habit, which an abused constitution reminds him every moment that he has made almost a physical necessity. The impossibility of legislating successfully against this evil, has been abundantly proven; we, however, can do something, as has been shown by the success in many instances of institutions for inebriates in other countries. Such a movement on the part of the profession in this country, will reflect upon them a lasting honor, and we all hope and pray it may be successful.

---

*Case of Imperforate Rectum.* Letter from Dr. Jones, of Charlotte, Monroe County, N. Y.

Editor of Buffalo Medical Journal.

Dear Sir: I have met with a very remarkable case in my practice, and if you think it worth publishing, you are at liberty to do so.

On September 18th, 1858, Mrs. Nelson was delivered, after a short labor, of a fine healthy boy, weighing nine and a-half pounds, plump and fat. The child seemed quite well for two days, nursed two or three times, slept well, and was quiet with the exception of occasional turns of vomiting a dark slimy substance, as infants frequently do at that age. After two days, we thought it should have its bowels evacuated, and therefore administered a laxative, which was speedily rejected. Another was given, but also rejected. The vomiting continued. bringing up a dark offensive substance, resembling meconium. We then commenced with enemata, which were not retained a moment. After having made repeated injections, failing to get any feces, I introduced a bougie about two and a-half inches, into the rectum, when I met with an obstruction. After ten days, the parents thought an operation might be performed to relieve the child, and Dr. Carpenter was called in. After examining the rectum, we passed a small gum-elastic catheter up the bowel, as we thought, about eight inches. Dr. C. thought it should remain for four or five hours. At the expiration of that time I removed it and found that it had coiled itself in the recium, which was very easily explained when the instrument became warm and soft. I judged from this, and previous examinations, that there was a permanent obstruction in the rectum. After twelve days suffering, the child keeping nothing on its stomach, and nothing passing its bowels, it died on the morning of the twelfth day.
